# Prolactin: A Key Immunoregulator in Viral Infections and Autoimmune Diseases

**DOI:** 10.1155/ije/2312675

**Published:** 2025-12-28

**Authors:** Mina Asadikaram, Saeed Bahrampour, Mahdis Rahimi Naiini, Abdollah Jafarzadeh, Clifford Rosen, Gholamreza Asadikaram

**Affiliations:** ^1^ Applied Cellular and Molecular Research Center, Kerman University of Medical Sciences, Kerman, Iran, kmu.ac.ir; ^2^ Kerman Medical Student Research Center, Kerman University of Medical Sciences, Kerman, Iran, kmu.ac.ir; ^3^ Department of Clinical Biochemistry, Afzalipour School of Medicine, Kerman University of Medical Sciences, Kerman, Iran, kmu.ac.ir; ^4^ Endocrinology and Metabolism Research Center, Institute of Basic and Clinical Physiology Sciences, Kerman University of Medical Sciences, Kerman, Iran, kmu.ac.ir; ^5^ Student Research Committee, Kerman University of Medical Sciences, Kerman, Iran, kmu.ac.ir; ^6^ Department of Immunology, Afzalipour School of Medicine, Kerman University of Medical Sciences, Kerman, Iran, kmu.ac.ir; ^7^ MaineHealth Institute for Research, Scarborough, Maine, 04074, USA

**Keywords:** autoimmune diseases, immune response, prolactin, viral infections

## Abstract

Prolactin (PRL) is secreted by various cells in the anterior pituitary gland, mammary glands, placenta, uterus, ovaries, testes, skin, adipose tissue, endothelial cells, immune system, and central nervous system. The expression and secretion of PRL are influenced by several factors such as suckling, thyrotropin‐releasing hormone (TRH), cytokines, dopamine, estrogen, and vasoactive intestinal polypeptide. It operates through a complex receptor, which is expressed in mammary gland cells, pancreatic beta cells, adipocytes, and immune cells. PRL is essential for various physiological functions, in particular milk production, breast development, metabolism, and immune regulation. Serum PRL levels fluctuate daily and can be affected by exercise, diet, and stress. Hyperprolactinemia is linked to autoimmune diseases and viral infections. In viral infections such as HIV, HCMV, HCV, and COVID‐19, PRL levels are often increased, which may influence the immune responses. PRL can modulate the activity of various immune cells, including T cells, B cells, natural killer cells, and macrophages, mounting an effective immune response against viral infections. Moreover, PRL influences the production of cytokines that mediate and regulate immunity and inflammation. PRL stimulates B cells to produce antivirus antibodies that are essential for neutralizing viruses and preventing their spread within the body. PRL levels, varying by sex and life stage, may affect immune responses and susceptibility to viral infections. Moreover, overexpression of PRL was indicated in various autoimmune diseases. Overall, PRL is a complex hormone with significant implications for endocrine function, immune regulation, and immune responses to viral infections, highlighting the need for further research into its diverse roles in health and disease. This review summarizes current knowledge of the immunomodulatory effects of PRL in human viral infections and possibly its contribution to the development of autoimmune diseases.

## 1. Introduction

### 1.1. Overview of Prolactin (PRL) and Its Biological Functions

**Figure 1:**
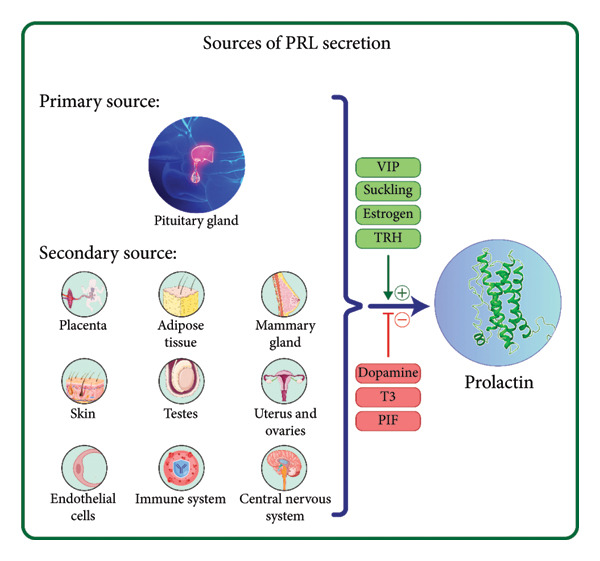
Sources of PRL secretion. The anterior pituitary gland is the primary source of prolactin, produced by lactotroph cells, with its secretion regulated by inhibitory factors such as dopamine, T3, and PIF and stimulated by factors including VIP, estrogen, TRH, and suckling. Secondary sources of prolactin include the placenta, adipose tissue, mammary glands, skin, testes, uterus, ovaries, endothelial cells, immune cells (e.g., lymphocytes), and the central nervous system. Abbreviations: PRL: prolactin; VIP: vasoactive intestinal peptide; TRH: thyrotropin‐releasing hormone; T3: triiodothyronine; PIF: prolactin inhibitory factor.

PRL is an endocrine protein hormone primarily secreted by lactotroph cells in the anterior pituitary gland. These secretagogues enhance PRL release through mechanisms involving calcium‐dependent exocytosis and transcriptional regulation [1]. Unlike other hormones of the anterior pituitary, PRL is not activated by hypothalamic‐releasing factors; instead, its secretion is inhibited by dopamine and a primary physiological PRL inhibitory factor (PIF) released from the hypothalamus. Additionally, triiodothyronine (T3) negatively impacts the human PRL gene promoter to influence PRL levels. In contrast, T3‐releasing hormone (TRH) acts as a PRL‐releasing factor (PRF), facilitating PRL release. Moreover, PRL exerts negative feedback on its secretion by activating tuberoinfundibular dopamine (TIDA) neurons through its interaction with the PRL receptor (PRLR) on these cells [2]. In addition to the pituitary gland, PRL can also be produced in various other tissues, including the mammary glands, placenta, uterus, ovaries, testes, skin, adipose tissue, endothelial cells, immune system, and central nervous system [3, 4]. The expression and secretion of PRL are influenced by several factors such as suckling, TRH, cytokines, estrogen, and vasoactive intestinal polypeptide (VIP) [5–7]. The expression of PRL in the pituitary gland is primarily governed by a balance of inhibitory and stimulatory molecules, including hormones, cytokines, and other factors that orchestrate a series of intracellular signaling events (Figure 1). Key signaling pathways involved in this process include cAMP/protein kinase A (PKA), phosphatidylinositol/Ca^++^/protein kinase C (PKC), and mitogen‐activated protein kinase (MAPK), which play significant roles in modulating PRL expression dynamics [8].

Serum PRL levels vary with the time of day and can be increased by exercise, a high‐protein diet, emotional stress, slumber, orgasm, prolactinoma, primary hypothyroidism, and adrenal insufficiency. The highest levels of PRL are observed during sleep and early in the morning, whereas the lowest levels occur during the day [9, 10]. The physiological concentration of fasting PRL is up to 25 ng/mL in females and up to 20 ng/mL in males [5].

PRL exerts endocrine, paracrine, and autocrine activities through binding to a complex receptor system, specifically PRLRs [5]. The PRLR belongs to the Type 1 cytokine receptor superfamily and is associated with biological processes such as cell differentiation, reproduction, and immune responses [4, 11]. It lacks an intrinsic kinase domain but has a region that associates with Janus kinase 2 (JAK2). This receptor family also includes receptors for leptin, erythropoietin, colony‐stimulating factor, and interleukin‐6 (IL‐6). PRLRs are found in various endocrine tissues and target organs, including the brain (with the highest concentration in the choroid plexus), liver, epithelial cells of the breast gland, prostate, placenta, seminal vesicles, and different kinds of immune cells [12].

The PRLR gene is located on Chromosome 5 and contains 11 exons [13]. Due to conserved homology, PRLRs can also function as receptors for human growth hormone (GH) and placental lactogen [5, 14]. Different PRLR isoforms can activate various signaling pathways, leading to alterations in cell survival, proliferation, and differentiation. PRL influences cell function, guides cell fate, regulates physiological homeostasis, and affects the tissue microenvironment [7, 15].

PRL plays an essential role in various biological functions [5]. PRL, also known as lactotropin, is responsible for milk production and breast development in females [5]. Additionally, it influences the regulation of sexual desire and maternal behavior [10]. PRL also affects metabolic homeostasis by maintaining the abundance of insulin‐producing cells (islets), regulating the hypothalamic energy center, promoting lipogenesis, balancing adipose tissue expansion, modulating insulin response in adipocytes, and affecting liver metabolism [16]. PRL also possesses regenerative properties for various tissues and cell types, including chondrocytes, oligodendrocyte precursor cells, and neural stem/progenitor cells [17].

**Figure 2:**
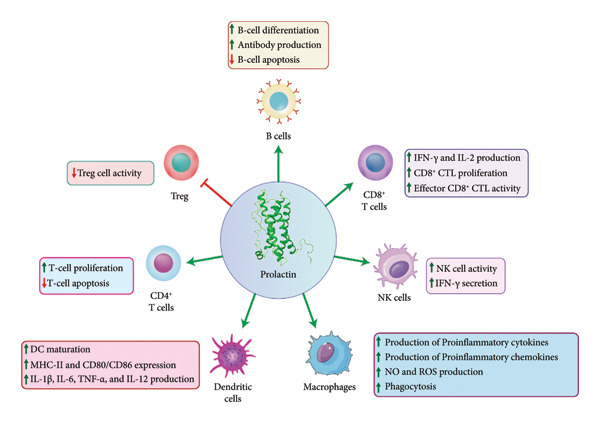
Immunomodulatory effects of prolactin on various immune cells. Prolactin modulates immune responses by enhancing B‐cell differentiation and antibody production, promoting CD4+ and CD8+ T‐cell proliferation and cytokine production, boosting NK cell activity, inducing dendritic cell maturation and cytokine release, and stimulating macrophage proinflammatory functions (green arrows indicate upregulation; red arrows indicate downregulation).

**Figure 3:**
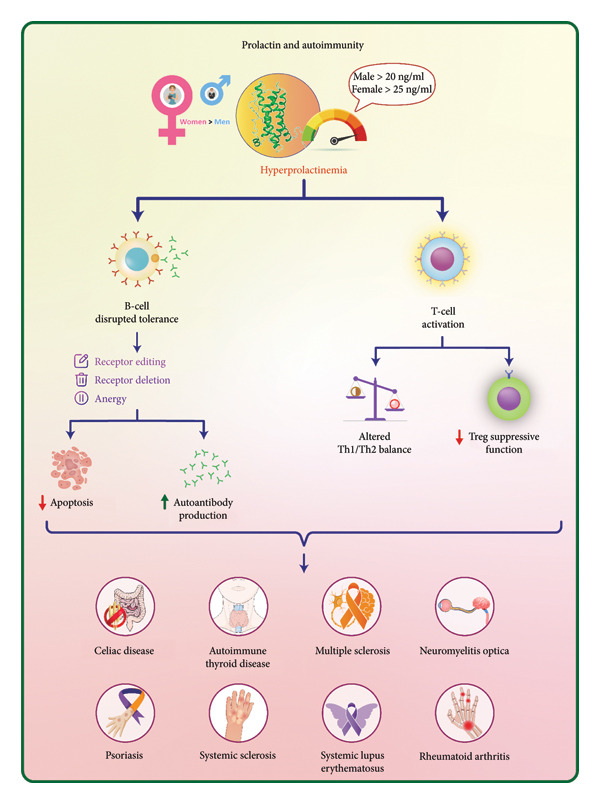
Role of prolactin in autoimmunity. Hyperprolactinemia, defined as prolactin levels above 20 ng/mL in males and 25 ng/mL in females, disrupts immune tolerance by impairing B‐cell receptor editing, deletion, and energy, leading to increased autoantibody production and apoptosis. It also activates T cells, shifts the Th1/Th2 cytokine balance, and reduces regulatory T‐cell (Treg) function. This immune dysregulation contributes to the development and activity of various autoimmune diseases—such as celiac disease, autoimmune thyroid disease, multiple sclerosis, neuromyelitis optica, psoriasis, systemic sclerosis, systemic lupus erythematosus, and rheumatoid arthritis—with a higher prevalence in women.

Structural analyses of PRL and its receptors have established their connection to the cytokine/hematopoietic family. PRL is a hormone primarily known for its role in lactation, but it also has powerful impacts on the immune system. Moreover, PRL plays a role in both proinflammatory and anti‐inflammatory functions [5]. As a cytokine derived from leukocytes, PRL plays a significant role in the immune system and is modulated by various cytokines through both autocrine and paracrine signaling mechanisms. In T lymphocytes, the expression of PRL mRNA is decreased by IL‐2, IL‐1β, and IL‐4, whereas IL‐10 and interferon‐γ (IFN‐γ) do not influence its expression [18]. Within the immune system, PRLR is expressed by various cells such as monocytes, macrophages, T lymphocytes, and B lymphocytes [12], indicating that PRL influences both innate and adaptive immune responses. This review provides an overview of the current understanding of PRL’s immunomodulatory role in human viral infections and possibly its contribution to the development of autoimmune diseases (Figures 2 and 3).

### 1.2. Significance of Immunoregulation in Viral Infections

Immunoregulation is a sophisticated mechanism that helps the immune system effectively combat viral infections while preventing potentially harmful inflammatory responses. During viral infections, the immune system must strike a delicate balance between eliminating the pathogen and protecting host tissues from damage [19, 20] (Table 1).

**Table 1:** Prolactin isoforms, sources, and regulatory factors.

Source/tissue	Isoform(s)	Molecular weight/characteristics	Biological activity	Stimulatory factors	Inhibitory factors
Anterior pituitary gland (lactotroph cells)	Full‐length PRL, glycosylated PRL	Full length: 23 kDa, 199 aa; glycosylated: ∼25 kDa, glycosylated	Full length: lactogenic, immunomodulatory; glycosylated: reduced receptor binding	Suckling, TRH [2], cytokines [8], estrogen [5–7], VIP [21]	Dopamine, PIF, T3 [2]
Mammary glands	Not specified (likely full‐length PRL)	—	—	Suckling and estrogen [5–7]	Dopamine [2]
Placenta	16‐kDa PRL, glycosylated PRL	16 kDa: 16 kDa, cleaved fragment; glycosylated: ∼25 kDa, glycosylated	16 kDa: antiangiogenic; glycosylated: reduced receptor binding	Cytokines and estrogen [5–7]	—
Uterus, ovaries, testes	Not specified	—	—	Estrogen [5–7]	—
Skin, adipose tissue, endothelial cells	Not specified	—	—	Cytokines [5–7]	—
Immune system (T/B cells, macrophages)	Full‐length PRL, delta‐PRL	Full length: 23 kDa, 199 aa; delta‐PRL: variable, truncated	Full length: immunomodulatory; delta‐PRL: immunomodulatory (context‐specific) [5, 22]	cAMP [23, 24], retinoic acid [25, 26], calcitriol [25]	IL‐2 [18], IL‐1β [18], IL‐4 [18], dexamethasone [1]

*Note:* Physiological PRL levels: up to 25 ng/mL (females) and 20 ng/mL (males). PIF, primary physiological PRL inhibitory factor; T3, triiodothyronine; TIDA, tuberoinfundibular dopamine.

Abbreviations: TRH, thyrotropin‐releasing hormone; VIP, vasoactive intestinal polypeptide.

## 2. PRL: Structure and Mechanism of Action

### 2.1. Molecular Structure of PRL

PRL has three isoforms produced by alternative splicing that can be distinguished chromatographically [10]. (i) Large (PRLR‐L), also known as big–big PRL or macroPRL, constitutes up to 1% of circulating PRL [10]. In humans, PRLR‐L is predominantly found in the placenta, adrenal gland, pituitary gland, and hippocampus [27]. (ii) Intermediate (PRLR‐I), also referred to as big PRL (with a molecular weight of 45–60 kDa), is primarily expressed in the placenta, adrenal gland, small intestine, and kidneys [10, 27]. (iii) Small (PRLR‐S), the most biologically active isoform, is monomeric and free, with a molecular weight of 23 kDa. This isoform constitutes up to 95% of serum PRL [10]. The expression of PRLR‐S has been documented in various organs, including the prostate, liver, and uterus [27]. The modified cytoplasmic domains of PRL are distinct, whereas the extracellular domains of the PRLRs are identical [27] (Table 1).

### 2.2. Regulation of PRL Expression in the Immune System

PRL is synthesized by various immune cells, including T and B lymphocytes, macrophages, thymocytes, mononuclear cells, and natural killer (NK) cells. In peripheral blood mononuclear cells (PBMCs), the production of PRL is predominantly linked to T lymphocytes [28, 29]. Unlike PRL expression in the pituitary gland, which is influenced by classical regulators such as Pit‐1, progesterone, estrogen, TRH, dihydrotestosterone, and insulin, lymphocyte‐derived PRL utilizes an alternative promoter that renders its expression independent of these factors [18, 29]. Instead, the expression of PRL in T lymphocytes is promoted by agents such as cAMP [23, 24], retinoic acid, and calcitriol [25, 26]. Conversely, this expression can be suppressed by dexamethasone and certain interleukins [1] (Table 1).

### 2.3. PRLR Expression

The expression of PRLR has been extensively studied across various immune cell types, including splenocytes, thymocytes, bone marrow cells, and PBMCs [30]. Within the immune system, PRL is primarily produced by T and B cells and macrophages [31, 32]. Notably, both PRL and its receptors are constitutively expressed in resting T cells, indicating a baseline level of readiness for immune modulation [4].

Beyond pituitary and immune sources, PRL can also be produced ectopically by several nonpituitary tumors. Early biochemical evidence demonstrated autonomous synthesis and secretion of PRL by human breast carcinoma and other neoplastic tissues independent of hypothalamic or pituitary control [33]. This ectopic PRL behaves similarly to pituitary PRL and may exert local autocrine and paracrine immunomodulatory effects within the tumor microenvironment, contributing to cellular proliferation and immune escape. Recognition of such nonpituitary sources therefore broadens the concept of PRL’s systemic and tissue‐specific immunoregulatory roles.

## 3. PRL and the Immune System

PRL plays a significant role in regulating the immune system through various mechanisms [22, 34]. PRL functions as a cytokine that enhances the growth and activity of various immune cells through both paracrine and autocrine mechanisms [35]. Additionally, PRL exhibits cytokine‐like activity in human mononuclear and polymorphonuclear leukocytes [36]. PRL influences both innate and adaptive immunity [35]. Although immune responses do not rely exclusively on PRL, this hormone can influence the phenotype and functions of cells in both the innate and adaptive branches of the immune system [17]. A balance between the two arms of the cellular immune response is crucial for the proper functioning of the immune system.

### 3.1. PRL Impacts on Innate Immunity

Innate immunity primarily relies on interferons (IFNs) and NK cell (NKC) activity in antiviral defense [37–39]. Upon PRLR engagement, PRL activates the canonical JAK2–STAT1/interferon regulatory factor‐1 (IRF‐1) axis, thereby enhancing IFN‐γ and IL‐2 transcription and promoting T cell [40, 41].

PRL also augments NK cell cytotoxicity by upregulating activating receptors and stimulating IFN‐γ production [42–45]. In vivo, these effects have been associated with reduced lymphocyte apoptosis in Chagas disease models [46] (Table 2).

**Table 2:** PRLR expression across immune cells.

Immune cell type	PRLR expression	Functional impact of PRL binding
T lymphocytes (CD4+ and CD8+)	Constitutive	Enhances IL‐2 [47], IFN‐γ production [5, 46, 48–50], proliferation [41, 51, 52]
B lymphocytes	Constitutive	Stimulates antibody production [4, 27, 53, 54] reduces apoptosis [46, 55, 56]
NK cells	Constitutive	Boosts activity [5, 42–44, 50, 51], IFN‐γ secretion [45], receptor expression [51]
Macrophages	Constitutive	Increases cytokine/chemokine secretion [57–59], phagocytosis [36]
Monocytes	Constitutive	Enhances inflammatory responses [51, 60, 61]
Splenocytes and thymocytes	Detected	Supports immune cell development [46]
PBMCs	Detected	Modulates T‐cell activity [4, 35]

Abbreviations: NK, natural killer; PBMC, peripheral blood mononuclear cells.

### 3.2. PRL Impacts on Adaptive Immunity

The adaptive immune response mainly involves B cells, which generate antivirus antibodies, as well as T cells, including CD4+ T cells that deliver helper signals to other immune cells and CD8+ cytotoxic T lymphocytes (CTLs) that eliminate virus‐infected cells [62–64]. PRL promotes B‐cell activation and differentiation, enhancing antibody production and survival through antiapoptotic and activation‐promoting mechanisms [53, 55, 56, 65].

During Chagas disease, PRL further enhances adaptive responses by increasing the proportion of splenic CD4+ T cells capable of producing IFN‐γ [46]. PRL acts as a mitogen, enhancing cellular survival in T cells [66] by promoting their longevity and increasing the production of key cytokines such as tumor necrosis factor‐alpha (TNF‐α), IFN‐γ, and IL‐2 in CD8+ T cells when treated with phorbol myristate acetate [47]. It should be noted that the cellular and humoral responses are initiated by Type 1 (Th1) and Type 2 (Th2) T‐helper cells, respectively [63, 67]. PRL plays a significant role in maintaining this balance between Th1 and Th2 responses. Additionally, PRL diminishes the suppressive functions of regulatory T cells (Treg) [68], thereby potentially enhancing immune responses [27].

Furthermore, PRLRs are also expressed on CD8+ CTLs [22]. Therefore, PRL can boost the cytotoxic capabilities of CD8^+^ CTLs, allowing them to more effectively kill virus‐infected cells [69]. However, it was reported that the physiological levels of PRL (12–25 ng/mL) boost CTL activity, whereas excessively high levels (200 ng/mL) can inhibit this response [69] (Table 2).

### 3.3. PRL Influence on Proinflammatory and Anti‐Inflammatory Cytokines

PRL engages classical cytokine signaling through the JAK2–STAT1/3/5 axis, which integrates upstream cues from immune and endocrine mediators [70–77]. This pathway balances pro‐ and anti‐inflammatory outputs: STAT1 activation favors IFN‐γ gene expression, whereas STAT3 mediates interleukin‐10 (IL‐10)‐driven anti‐inflammatory effects in macrophages [73–77]. Beyond signaling, PRL modulates cytokine secretion across multiple immune compartments. It promotes the release of IFN‐γ, interleukin‐12 (IL‐12), and IL‐10 in peripheral blood cells [78] and enhances macrophage chemokine production including macrophage inflammatory protein‐1α (MIP‐1α), monocyte chemoattractant protein‐1 (MCP‐1), IFN‐γ‐inducible protein 10 (IP‐10), and C–C motif chemokine ligand 5 (CCL5) that coordinate leukocyte recruitment [57]. PRL also elevates reactive oxygen species (ROS) and boosts phagocytic and cytotoxic capacity in tumor‐associated macrophages [58, 59]. In granulocytes, PRL upregulates inducible nitric oxide (NO) synthase (iNOS) and IRF‐1 expression, supporting antimicrobial activity [36].

Collectively, PRL exerts dual immunoregulatory roles by simultaneously amplifying protective inflammatory pathways while activating counter‐regulatory signals that prevent excessive tissue injury (Table 2).

### 3.4. PRL‐Related Modulatory Impacts on the Circadian System, Sleep–Wake Cycle, and Immune Function

Research indicates that the composition and levels of immune system cellular components fluctuate according to circadian rhythms. Notable examples include IgE‐dependent activation by mast cells [79], expression of cell‐adhesion molecules, activation of NKCs [80], and worsening of clinical symptoms related to rheumatoid arthritis (RA) in the morning, correlating with the daily rhythm of the proinflammatory cytokine IL‐6 [81]. The immune function is significantly affected by the various changes that occur during sleep. Notably, sleep leads to a marked increase in the production of IL‐12 by myeloid dendritic cell precursors, suggesting a promotion of Th1 immune responses. Conversely, sleep results in a reduction in both plasmacytoid dendritic cells and T‐cell populations, although it does not impact the production of IFN‐α. Additionally, sleep substantially decreases certain monocyte subpopulations, specifically CD14^+^ and CD16^+^ cells, likely due to their margination associated with a decrease in catecholamine levels during sleep [82]. The levels of undifferentiated naive T cells and the production of proinflammatory cytokines reach their highest levels during the initial hours of sleep.

In contrast, the circulatory immune cells with immediate effect or functions, along with the activity of anti‐inflammatory cytokines, peak during periods of wakefulness. Sleep also promotes the movement of T cells from the bloodstream into tissues and their subsequent redistribution to lymph nodes. Overall, sleep enhances the production of certain cytokines, such as IL‐12, which facilitate interactions between antigen‐presenting cells (APCs) and T‐helper cells [83].

Nonrapid eye movement (NREM) sleep is thought to play a crucial role in the transfer of antigenic information from APCs to antigen‐specific Th cells. This process may enhance immunological memory following vaccination [84], as evidenced by observations that sleep postvaccination boosts immune responses. The daily pattern of circulating PRL, which peaks during rest periods in mammals, suggests that PRL might contribute to the improved immune response associated with sleep [1].

## 4. PRL and Autoimmunity

Altered levels of PRL, which may favor either Th1 or Th2 dominance, are often linked to autoimmune diseases [67]. The impact of hyperprolactinemia on the immune system varies based on its duration. Acute exposure to elevated PRL levels can increase inflammation during stress, whereas chronic hyperprolactinemia tends to have an immunosuppressive effect [85]. The effects of PRL on the immune response are dual and primarily depend on its concentration. Higher levels of PRL tend to suppress the immune system, whereas lower levels promote immune stimulation [86, 87]. Hyperprolactinemia, characterized by elevated PRL levels, is primarily viewed as a pathological condition, particularly when excluding physiological instances such as pregnancy. This condition has been linked to various autoimmune diseases, including systemic lupus erythematosus (SLE) [88], multiple sclerosis (MS) [89], and RA [90], with a notable prevalence among women, especially during pregnancy [91]. Research indicates that women typically have higher baseline PRL levels compared to men, which may contribute to the increased incidence of these autoimmune disorders in females [91]. Therapeutically, bromocriptine—a dopamine agonist that reduces PRL levels—has shown promise in managing these autoimmune conditions. By lowering PRL concentrations, bromocriptine may help suppress the autoimmune processes linked to hyperprolactinemia [91]. It is noteworthy that immunostimulatory PRL can display an immunosuppressive response at relatively high doses under specific conditions [91]. PRL has been demonstrated to have a significant role in inhibiting apoptosis in lymphocytes [92]. In BALB/c mice that carry a transgene for the heavy chain of a pathogenic anti‐DNA antibody [93], inducing moderate hyperprolactinemia (resulting in a twofold increase in serum PRL levels) enhances the population of autoreactive B cells exhibiting a follicular phenotype. This increase in B cells leads to their activation, which subsequently triggers the production of anti‐DNA antibodies and IgG deposition in the kidneys [55]. The induction of hyperprolactinemia enhances autoreactivity by disrupting B‐cell tolerance. This occurs through three primary mechanisms that govern the induction of B‐cell tolerance: receptor‐mediated deletion, receptor editing, and anergy [55]. Although there is evidence suggesting that PRL plays an immunomodulatory role, studies have demonstrated that the development of the immune system remains unchanged in both PRL knockout and PRLR knockout mice [94].

### 4.1. PRL and Some Immunologic Disorders

#### 4.1.1. PRL and Systemic Sclerosis

Systemic sclerosis is a complex systemic autoimmune disease characterized by progressive fibrosis, microvascular damage, and immune system dysregulation, resulting in excessive collagen and extracellular matrix accumulation within the skin and multiple internal organs [95]. Elevated PRL levels have been documented in a range of 13%–59% of patients diagnosed with systemic sclerosis, suggesting a potential hormonal involvement or endocrine dysregulation in this complex autoimmune connective tissue disorder [96]. A robust statistical relationship was discovered linking hormonal concentrations directly to the progression and intensity of skin hardening (sclerosis), pulmonary complications, and cardiovascular system manifestations ([97]{La Montagna, 2001 #313, [98]). The pathogenesis of PRL elevation in this disease is attributed to multiple mechanisms, including augmented lymphocyte secretory activity, heightened central dopaminergic tone, and pharmacological induction primarily through antidepressant and prokinetic medications [99]. Pregnancy does not inherently worsen the underlying disease, although isolated cases have been documented involving women experiencing organ dysfunction, particularly pulmonary hypertension and extensive skin fibrosis ([100]{Tincani, 2016 #317). In conclusion, the research demonstrated a statistically significant correlation between PRL levels and the progression and intensity of the underlying disease pathology (Table 3).

**Table 3:** PRL in autoimmune diseases.

Autoimmune disease	PRL level change	Key effects	Therapeutic insight
Systemic sclerosis	Elevated (13%–59%) [96, 99]	Correlates with skin sclerosis, pulmonary issues [97, 98, 100]	—
Multiple sclerosis (MS)	Elevated (mild–moderate) [89, 101, 102]	Increases B‐cell autoreactivity [103], varies by gender [104]	—
Celiac disease	Elevated (untreated) [105–107]	Correlates with mucosal atrophy [107, 108], reduced by GFD (125)	Gluten‐free diet reduces PRL [105–107]
Systemic lupus erythematosus (SLE)	Variable (elevated in some) [109, 110]	Enhances NK activation and autoantibody production [54, 111]	Bromocriptine may suppress [91]
Autoimmune thyroid disease	Elevated (20% and 90% in HT) [112]	Linked to hypothyroidism, low cortisol [112]	Dopamine agonists under study [112]
Neuromyelitis optica (NMO)	Elevated during attacks [113]	Suggests an immunomodulatory role [113]	—
Rheumatoid arthritis (RA)	Elevated in synovial tissue [90]	Increases in TNF‐α correlate with disease activity [61, 114, 115]	High‐dose PRL protective in models [116]
Psoriasis	Elevated (debated) [117, 118]	Promotes keratinocyte proliferation [48, 119], IFN‐γ [48, 120]	Treatment reduces PRL levels [117, 118]
Idiopathic granulomatous mastitis (IGM)	Elevated in hyperprolactinemic states [121, 122]	Stimulates macrophage and lymphocyte activation, promoting granuloma formation	Dopamine agonists normalize PRL and prevent recurrence [121, 122]

Abbreviations: GFD, gluten‐free diet; HT, Hashimoto’s thyroiditis; TNF‐α, tumor necrosis factor‐alpha.

#### 4.1.2. PRL and MS

MS is a complex autoimmune disease affecting the central nervous system, characterized by an intricate interplay of immune dysregulation, where inflammatory processes lead to demyelination and neuronal damage through the activation of various immune cells in the brain and peripheral blood [123]. The PRL gene is situated on the short arm of chromosome 6p, positioned near the HLA‐DRB1 region and the TNF‐α gene, both of which play significant roles in modulating PRL gene expression [124]. The relationship between PRL levels in MS is complex, with potential dual roles involving both neuroprotective mechanisms and proinflammatory effects that could impact disease progression [125]. A comparative research investigation demonstrated that MS patients of Asian descent exhibit significantly higher serum PRL levels compared to their Western counterparts [101]. A research investigation revealed that female patients with relapsing‐remitting MS (RRMS) exhibited heightened PRL concentrations in both serum and cerebrospinal fluid, whereas male patients demonstrated standard PRL levels [104]. The studies demonstrate significant racial and gender influences on PRL secretion and its implications in MS pathogenesis. In MS, PRL triggers increased CD40 surface expression on B cells, which subsequently amplifies their autoreactive characteristics [103]. Elevated PRL concentrations have been observed in MS patients [89, 102]. Increased PRL production has been associated with the corticospinal MS variant, which is characteristic of Asian populations [101]. Emerging evidence proposes that hyperprolactinemia may play a contributory role in the underlying pathogenetic mechanisms of autoimmune demyelinating neurological disorders affecting the central nervous system. Researchers have discovered elevated PRL levels in neuromyelitis optica (NMO) and clinically isolated syndrome (CIS) patients, indicating that altered PRL production is not unique to MS but may be a broader characteristic of autoimmune demyelinating disorders [113]. Research indicates that MS patients experienced mild‐to‐moderate hyperprolactinemia, compared to healthy subjects [89, 102, 126]. Some studies have not found a significant association between PRL levels, oligoclonal band status, and disease duration [104, 127–129]. Kira et al. discovered that among eight patients with hyperprolactinemia, four were found to have diencephalon–hypothalamic lesions, leading them to hypothesize that MS‐related lesions in the hypothalamus might disrupt dopamine release, the PIF [102] (Table 3).

#### 4.1.3. PRL and Celiac

Celiac disease represents an immune‐driven disorder characterized by an inflammatory small intestinal condition that triggers a permanent adverse reaction to gluten‐containing proteins derived from wheat and related cereal grains, resulting in chronic digestive and systemic complications [130–132]. Scientific investigations have elucidated the complex mechanisms by which PRL influences adaptive hyperplastic and hyperfunctional processes within intestinal mucosal tissue ([108]{Mainoya, 1978 #341). The presence of PRLRs in the human small intestine challenges previous reports suggesting that PRL is not trophic to the small intestine, indicating potential physiological interactions and signaling mechanisms that warrant further investigation [133]. Elevated serum PRL levels observed in untreated celiac disease patients may potentially indicate a primary immunological mechanism that triggers intestinal mucosal damage and subsequent clinical manifestations. Intestinal mucosal injury can potentially modulate the expression and functionality of PRLRs within the human gastrointestinal epithelial lining [133]. Compared to children with celiac disease following a gluten‐free diet, those who continue to consume gluten demonstrate markedly elevated PRL levels [105, 106, 134]. PRL serum levels demonstrated a positive correlation with three key parameters: the extent of disease activity, the severity of mucosal atrophy, and the concentration of antiendomysial antibodies in the serum [107]. A gluten‐free diet appears to directly influence hormone levels, as demonstrated by the concurrent reduction in PRL and antitransglutaminase antibodies, indicating a potential metabolic interrelationship [107] (Table 3).

#### 4.1.4. PRL and SLE

SLE is a chronic autoimmune disease in which the body’s immune system mistakenly attacks its tissues, causing widespread inflammation and potential damage across multiple organ systems [135]. SLE patients exhibit variable plasma/serum PRL levels across different geographical regions, with some populations demonstrating consistently elevated concentrations compared to healthy controls [109]. In SLE, there is a significant increase in NKCs expressing PD1 and T‐cell‐induced negative regulator TIM‐3, which correlates positively with elevated inflammatory markers (erythrocyte sedimentation rate and C‐reactive protein [CRP]) and anti‐dsDNA autoantibody levels, indicating a direct relationship with disease activity and severity [136]. Elevated PRL levels stimulate NKC activation, thereby promoting inflammatory processes and potentially exacerbating SLE, which underscores the critical role of PRL in disrupting NKC development and potentially contributing to autoimmune pathogenesis [111]. PRL stimulates transcriptional regulation through IRF‐1 activation in monocytes, which substantially enhances the expression of multiple genes central to the SLE interferon signature, thereby reinforcing PRL’s critical role in SLE pathogenesis [60]. Alemán‐García et al. discovered that PRL enhanced both the quantity and functional activation of IL‐21‐producing OX40‐positive T follicular helper cells, which subsequently disrupted immune tolerance mechanisms by stimulating germinal center formation, promoting the development of autoreactive plasma cells, and ultimately triggering autoantibody production [54]. In patients with SLE, an elevated proportion of CD4+ OX40+ T cells serves as a potential biomarker correlating with heightened disease activity [137]. Although a definitive causal link between PRL secretion and neutrophil dysfunction in SLE remains unestablished, research indicates that PRL can modulate neutrophil function by significantly influencing their phagocytic capabilities and intracellular pathogen elimination mechanisms [138]. Neutrophil migration appears compromised or impaired in patients experiencing hyperprolactinemia [139]. Al‐Bayyoumy and colleagues observed no statistically significant variation in PRL concentrations between patients with active and inactive disease states before initiating treatment [140]. Pacilio et al. demonstrated a direct correlation between elevated PRL levels (hyperprolactinemia) and neurological manifestations involving the central nervous system [141]. The intricate pathophysiological relationship between elevated PRL levels and increased IL‐6 concentrations in neuropsychiatric lupus patients provides compelling evidence for a bidirectional communication mechanism between the neuroendocrine and immune regulatory networks [110] (Table 3).

#### 4.1.5. PRL and Autoimmune Thyroid Disease (AITD)

AITDs primarily encompass two clinical presentations: Graves’ disease and Hashimoto’s thyroiditis, both characterized by immune‐mediated attacks on the thyroid gland [142]. A comprehensive analysis revealed that hyperprolactinemia occurred in 20% of patients with AITD, with a notably higher prevalence (double the frequency) among those with hypothyroidism. Furthermore, approximately 90% of Hashimoto’s thyroiditis patients exhibited significantly elevated PRL levels, which were concurrently associated with reduced cortisol concentrations [112]. The potential therapeutic role of dopamine agonists in managing AITD remains an area of ongoing research and scientific investigation (Table 3).

#### 4.1.6. PRL and NMO

NMO is a rare autoimmune disease that targets the central nervous system, causing inflammation and damage specifically to the optic nerves and spinal cord [143]. Elevated PRL levels during MS and NMO attacks, particularly the more pronounced elevations observed during myelitis episodes, suggest a potential immunomodulatory role for PRL in the pathogenesis of autoimmune demyelinating diseases [113] (Table 3).

#### 4.1.7. PRL and RA

RA distinguishes itself among autoimmune diseases through its persistent inflammatory process, which not only targets joint structures but also impacts multiple systemic bodily systems [144]. Patients with active inflammatory arthritis, particularly RA, exhibit elevated PRL expression within their synovial tissue compared to healthy controls [90]. Monocytes derived from RA patients demonstrate an increased release of TNF‐α when exposed to PRL [61]. In a rodent model of inflammatory arthritis triggered by intra‐articular cytokine injection, both high‐dose PRL and the dopaminergic antagonist haloperidol demonstrated comparable protective effects, mitigating joint damage by suppressing chondrocyte programmed cell death [116]. The authors argue that PRL primarily functions as a catalytic mechanism in the pathogenesis of autoimmune disorders, suggesting its role as an initial inflammatory instigator ([114]{Ewerman, 2020 #378). Although some researchers argue that the mechanism primarily sustains inflammatory processes, others contend that it plays a more complex role in inflammatory activity [145]. In RA, compromised hypothalamic–pituitary–adrenal axis function coupled with sympathetic nervous system dysregulation can exacerbate stress‐related inflammatory disease progression [146]. During pregnancy, placental steroid hormones that induce hyperprolactinemia play a significant role in modulating RA remission mechanisms [147, 148]. The study revealed a positive correlation between varying PRL concentrations and increased clinical manifestations, including fatigue, morning stiffness, elevated disease activity scores, and glycemic irregularities in RA patients [115] (Table 3).

#### 4.1.8. PRL and Psoriasis

Psoriasis is a chronic autoimmune skin disorder primarily mediated by T cells, where environmental factors, potentially including viral antigens, trigger T‐cell activation and cytokine production. These cytokines subsequently promote keratinocyte proliferation and the expression of adhesion molecules in dermal blood vessels. Among the various mediators influencing keratinocyte behavior, PRL has been identified as having significant effects on epithelial cells, lymphocytes, and keratinocytes [117]. PRL appears to be implicated in the development of psoriasis and may serve as a biological marker for the disease’s activity. This hormone could be involved in both the causative mechanisms and the consequences of psoriasis pathology [117]. Keen and Hassan demonstrated that serum PRL levels were significantly elevated in patients compared to the control group [117]. PRL promotes the proliferation of cultured human keratinocytes and increases the production of VEGF in vitro [119, 149]. PRL may play a significant role in the development of psoriasis by promoting several key processes. It stimulates the proliferation of keratinocytes, enhances the production of IFN‐γ by T lymphocytes, and fosters angiogenesis [48, 49], all of which are crucial in the pathogenesis of this chronic inflammatory skin disease. Additionally, PRL’s inhibition of T‐suppressor cell functions could further facilitate the formation of psoriatic plaques [150]. Moreover, PRL amplifies the transcription and secretion of critical chemokines such as CXCL9, CXCL10, and CXCL11 in response to IFN‐γ, which encourages the infiltration of Th1 T‐helper cells into psoriatic lesions [120]. Studies on serum PRL levels in psoriasis have yielded inconsistent findings. Some research indicates that psoriatic patients exhibit significantly elevated serum PRL levels compared to healthy controls, with evidence suggesting local production of PRL in lesional skin that may contribute to the disease’s pathology [117, 118]. However, other studies have found no significant difference in serum PRL concentrations between psoriatic patients and healthy individuals, indicating that the role of PRL in psoriasis requires further investigation [151, 152]. PRL, often referred to as a stress hormone, can see elevated levels during times of psychological stress. This increase may worsen psoriasis symptoms. Notably, this phenomenon is especially pronounced during the postpartum period, when physiological hyperprolactinemia occurs. [153]. Psoriasis treatment seems to have an impact on serum PRL levels. Various therapeutic approaches, including both topical and systemic treatments, have been associated with a notable decrease in serum PRL levels among patients undergoing psoriasis treatment. Some studies indicate that after treatment, there is a significant reduction in serum PRL levels, suggesting a correlation between treatment effectiveness and hormone regulation in psoriatic patients [117, 118].

In conclusion, the connection between PRL and psoriasis is intricate and somewhat debated. Some studies indicate that elevated PRL levels may be present in patients with psoriasis and could contribute to worsening the condition. However, other research contradicts these findings. To better understand the role of PRL in the development of psoriasis, as well as its potential as a therapeutic target or biomarker for disease activity, further investigation is essential (Table 3).

#### 4.1.9. PRL and Idiopathic Granulomatous Mastitis (IGM)

IGM represents one of the most relevant tissue‐specific inflammatory disorders associated with hyperprolactinemia. Recent clinical observations describe strong associations between elevated serum PRL states including postpartum and lactational periods, prolactinomas, and antipsychotic‐induced hyperprolactinemia and the onset or recurrence of IGM [121]. PRL appears to contribute to IGM through its cytokine‐like actions on macrophages and lymphocytes, promoting proinflammatory cytokine release and granuloma formation within breast lobules. The condition often resolves following normalization of PRL levels with dopamine agonists such as bromocriptine, underscoring a causal endocrine–immune link. A large retrospective cohort confirmed that the difference between pre‐ and post‐treatment PRL values was an independent risk factor for recurrence in IGM patients, emphasizing the clinical importance of monitoring PRL during and after treatment [122]. Altogether, IGM illustrates how PRL‐mediated immune dysregulation can manifest as localized granulomatous inflammation in hormone‐responsive tissues.

## 5. Viral Infections in Endocrinology

Viruses are small obligatory intracellular parasites that possess either single‐ or double‐stranded RNA or DNA genomes [154]. They cannot replicate independently and must enter host cells, such as those of bacteria, algae, fungi, plants, insects, and vertebrates, to initiate viral replication. This entry process begins with the recognition of specific receptors on the host cell surface. Once inside, numerous structural and functional changes occur in the infected cell to facilitate viral replication [155]. The antiviral immune system plays a crucial role in defending the host organism against viruses. However, this immune response can also lead to inflammation and damage to nearby uninfected cells [156, 157]. The endocrine system is a sophisticated network of hormone‐producing cells and organs that is essential for maintaining homeostasis and regulating the immune response to infections. Numerous epidemiological and clinical studies have identified various endocrine and metabolic dysfunctions that can occur following viral infections, including those caused by human immunodeficiency virus type 1 (HIV‐1) [158], coxsackieviruses B (CVB) [159], and severe acute respiratory syndrome coronaviruses (SARS‐CoV) [154, 160].

### 5.1. PRL in Viral Infections

#### 5.1.1. Changes in the PRL Levels During Viral Infections

The role of PRL in the pathophysiology of viral infections involves its participation in viral entry and replication processes, as well as the stimulation of PRL secretion through inflammatory signaling pathways. This mechanism accounts for the elevated serum levels of PRL observed in various viral diseases. Notably, PRL possesses anti‐inflammatory properties, suggesting that the increase in serum PRL levels during viral infections may serve as a compensatory response to counteract inflammation and restore homeostasis [4]. PRL and its receptors are crucial in modulating both innate and adaptive immune responses, particularly influencing the growth and activation of T lymphocytes. This regulatory role has been observed during the COVID‐19 pandemic and in other severe acute viral infections [156]. Research indicates that certain viruses may exploit PRLR as entry points into host cells, revealing a complex evolutionary interplay between hormones and viral pathogens. This phenomenon suggests that the evolution of PRL and its receptors is not solely for endocrine functions but may also be shaped by viral interactions [161]. Beyond its function in boosting immune responses, PRL has exhibited antiviral properties in specific contexts. For example, research indicates that PRL can influence the clearance of avian leukosis virus (ALV‐J) viremia in animal models. This finding suggests that PRL may not only inhibit certain viral infections directly but also aid in their resolution [162].

In summary, PRL significantly influences immune responses during viral infections. It not only modulates these responses but may also serve as a receptor for certain viruses. PRL exhibits a dual role, possessing both proinflammatory and anti‐inflammatory properties, which positions it as a crucial factor in the pathophysiology of viral diseases.

##### 5.1.1.1. Correlation Between PRL Levels and Disease Severity

Acute viral infections trigger an increase in PRL levels through the stimulation of specific cytokines. Inflammatory cytokines such as IL‐1, IL‐2, and IL‐6 play a crucial role in promoting PRL production during viral infections [51]. Viral infections such as HIV can directly diminish dopaminergic tone, consequently leading to elevated PRL levels [163]. PRLRs are expressed on macrophages, monocytes, lymphocytes, and NKCs, enabling PRL to engage these immune cells through receptor binding, which subsequently triggers intracellular signaling cascades that promote immune cell proliferation, differentiation, and enhanced cellular survival [51]. PRL counteracts the immunosuppressive mechanisms of TNF‐α and TGF‐β, thereby enhancing immune system responsiveness [85].

**Figure 4:**
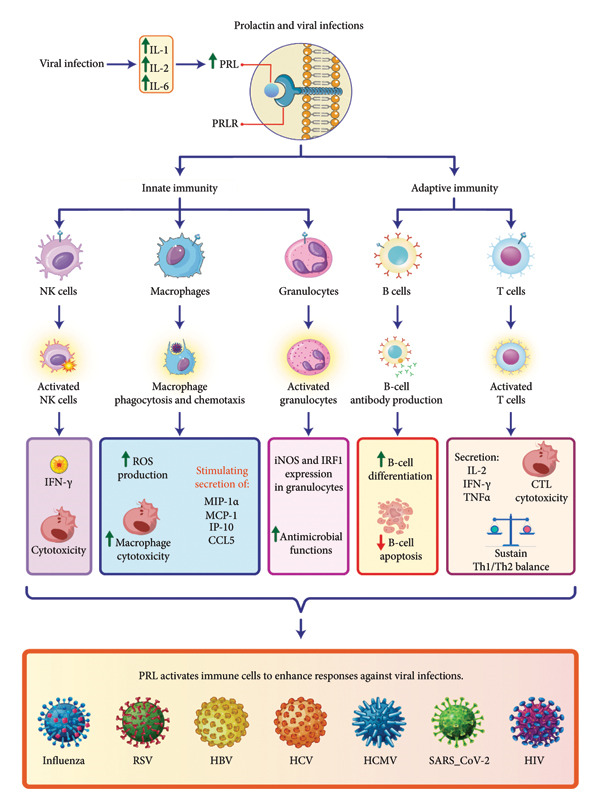
The role of prolactin in immune response to viral infections. PRL activates immune cells to fight viral infections via innate and adaptive immunity. In innate immunity, it boosts NK cells, macrophages, and granulocytes, increasing ROS production, macrophage cytotoxicity, and cytokine release (IFN‐γ, MIP‐1α, and IP‐10). In adaptive immunity, PRL enhances B‐cell differentiation for antibody production, T‐cell activation, and secretion of IL‐2, IFN‐γ, and TNF‐α, while promoting CTL cytotoxicity, Th1/Th2 balance, and B‐cell apoptosis. These actions target viruses such as influenza, RSV, HBV, HCV, HCMV, SARS‐CoV‐2, and HIV.

In summary, elevated PRL levels serve as an immunomodulatory agent during acute infectious processes, dynamically influencing immune system responses and inflammatory mechanisms. PRL has a complex relationship with viral disease severity, showing both proinflammatory and anti‐inflammatory effects across different viral infections (Figure 4).

In this section, we deal with it by classifying diseases.

##### 5.1.1.2. Immunomodulatory Effects of PRL in HIV Infection

The pituitary gland is influenced by various stages of HIV infection. Research indicates that the mean basal serum levels of GH, PRL, and testosterone are comparable between HIV‐positive individuals (regardless of AIDS status) and healthy controls. However, those with AIDS exhibit elevated basal serum concentrations of thyroid‐stimulating hormone (TSH), luteinizing hormone (LH), adrenocorticotropic hormone (ACTH), and cortisol. Furthermore, poststimulation peak levels of GH, PRL, TSH, and ACTH are also significantly higher in this group, suggesting increased pituitary gland activity [164]. PRL levels are elevated in individuals diagnosed with HIV, or hyper‐PRL emia, and have been associated with HIV infection. Elevated PRL levels enhance the activity of immune cells, and it is hypothesized that PRL levels may influence prognosis and pave the way for innovative therapeutic approaches [165]. Hyper‐PRL emia is frequently observed in patients with HIV, affecting approximately 20% of male individuals who are in a stable clinical condition. This elevation in PRL levels is not correlated with factors such as antiretroviral therapy (ART), metabolic disturbances, liver disease, or viral load [166]. Studies indicate that serum PRL levels in AIDS patients are significantly elevated compared to those of seronegative homosexual men and healthy controls. This increase in PRL is noteworthy because it is associated with lymphocyte activation and lymphoproliferation, processes crucial for immune response [52]. Research indicates that hyper‐PRL emia is prevalent among HIV‐infected individuals, particularly during the onset of secondary infections, but it does not correlate with metabolic disorders, liver dysfunction, viral load, or the use of ART [4, 5]. A prospective study involving 192 men diagnosed with HIV infection revealed that hyper‐PRL emia occurs in 21.4% of these patients. Notably, this condition is correlated with higher CD4^+^ counts, indicating a potential relationship between PRL levels and immune function in HIV‐infected individuals [166]. High levels of PRL have been proposed as a potential cause of hypogonadism in HIV patients, primarily due to PRL’s inhibitory effect on the release of gonadotropin‐releasing hormone from the hypothalamus. However, contrasting evidence suggests that hypogonadism associated with hyperprolactinemia in these patients may not significantly correlate with the suppression of gonadotropin release [167] (Table 4).

**Table 4:** PRL levels and effects on viral infections.

Viral infection	PRL level change	Immunomodulatory effects	Disease severity correlation
HIV	Elevated [163, 165] (20%–21.4% of males) [166]	Enhances lymphocyte activation [52, 156] and increases CD4+ counts [166]	Higher severity of secondary infections [52, 166]
HCMV	Elevated [168]	Increases PRLR expression [168–170], promotes inflammation [168]	Notable in immunocompromised states [161, 168, 171]
HCV	Elevated (males > females) [61, 172]	Induces PREB [172, 173], disrupts B‐cell tolerance [174, 175], causes thrombocytopenia [175, 176]	Linked to HCC and liver dysfunction [177–179]
HBV	Elevated [180–182]	Activates NK cells [183], correlates with cirrhosis severity [181, 184]	Predicts mortality (> 50 ng/mL in hepatic encephalopathy) [184, 185]
RSV	Elevated in severe cases [186–189]	Modulates CD4+ T cells [190, 191], reduces Treg suppression [191]	Higher levels in ICU‐admitted infants [186–189]
SARS‐CoV‐2 (COVID‐19)	Elevated [4, 51, 192–197]	Dual role: pro‐ and anti‐inflammatory cytokine effects [4, 50, 193]	Correlates with CRP levels, severe cases [50]
Influenza (H1N1)	Downregulated (mammary) [198, 199]	Potential antiviral metabolite [199]	Limited data on severity

*Note:* HCMV: human cytomegalovirus; HCV: hepatitis C virus; HBV: hepatitis B virus; CRP: C‐reactive protein; PREB: PRL regulatory element binding.

Abbreviations: HE, hepatic encephalopathy; RSV, respiratory syncytial virus.

##### 5.1.1.3. Immunomodulatory Effects of PRL in Human Cytomegalovirus (HCMV) Infection

HCMV is a prevalent DNA herpesvirus that affects approximately 60%–90% of adults globally. Its prevalence is notably higher among individuals from non‐Caucasian backgrounds and those with lower socioeconomic status [171]. HCMV is a common virus that can lead to significant health issues, particularly in immunocompromised individuals. An intriguing link exists between HCMV and PRL, particularly through the involvement of cyclophilin A (CypA) and cyclosporine, an immunosuppressive medication commonly used in transplant patients. Research has highlighted a functional interaction between CypA and the PRLR, demonstrating that CypA can modulate the activity of this receptor [169, 170]. Furthermore, HCMV infection promotes the expression of PRLRs in ovarian cancer by activating inflammatory signaling pathways such as nuclear factor kappa‐light‐chain‐enhancer of activated B cells (NF‐κB) and MAPK. This activation leads to the suppression of anti‐inflammatory pathways, which further enhances viral replication and exacerbates inflammatory responses. Both HCMV and PRL may utilize similar immunological pathways, contributing to an inflammatory environment that supports viral activity and impacts cancer progression [168]. Wallis demonstrated that PRLRs can function as both a receptor and an entry point for viral communication between CMV and host cells, as well as for other viruses such as Rubella [161].

In summary, the interaction between HCMV and PRL reveals a complex mechanism that may affect immune responses and tumor behavior. This intriguing relationship calls for further investigation in the fields of virology and oncology (Table 4).

##### 5.1.1.4. Immunomodulatory Effects of PRL in Hepatitis C Infection

Hepatitis C virus (HCV) is classified as a single‐stranded, positive‐sense RNA virus belonging to the Flaviviridae family [200]. Studies conducted both in vitro and in vivo have identified PRL regulatory element binding (PREB) as a novel cofactor in the infection process of HCV. HCV infection induces the expression of PREB, which subsequently facilitates the replication of HCV RNA by contributing to the formation of specific compartments necessary for viral replication [172]. Autoimmunity frequently occurs in individuals infected with HCV. The dysfunction of B cells in these patients may stem from their interaction with HCV, which alters B‐cell functions, leading to polyclonal activation and an increase in CD5+ B cells. Research indicates that hypergammaglobulinemia (HPRL) was observed in 10.1% of patients with HCV, and this condition was found to be independent of the presence of cryoglobulinemia or nonorgan‐specific autoantibodies [174]. A study indicates that HPRL is found in a subset of patients suffering from thrombocytopenia related to HCV infection, suggesting it may play a role in the disease’s pathogenesis. Consequently, treatments targeting PRL could be beneficial for these patients [176]. A prospective study indicated that patients with HCV exhibited elevated serum PRL levels compared to control groups. This increase was attributed to the induction of PRL mRNA in PBMC by HCV [4]. Hyper‐PRL emia is indeed linked to HCV infection; however, this connection does not extend to the extrahepatic manifestations typically associated with HCV, such as autoimmune disorders [201]. The findings indicate that serum PRL levels are elevated in males infected with HCV compared to healthy males. Furthermore, HCV infection appears to stimulate the expression of PRL mRNA in PBMCs, with this effect being more pronounced in males than in females [35]. High levels of PRL serum in HCV infection may lead to immune‐mediated thrombocytopenia by disrupting B‐cell tolerance, which, in turn, induces the production of autoantibodies [175]. Kong et al. conducted an in vitro study revealing that the PREB functions as a novel cofactor in HCV infection. Their findings indicate that HCV induces the expression of PREB, which subsequently enhances the replication of HCV RNA by facilitating the formation of specialized replication compartments within the host cell [173]. Hepatocellular carcinoma (HCC) ranks as the fifth most common cancer globally, with approximately 900,000 new cases diagnosed each year [177]. The incidence of HCC has been observed to double in certain regions, particularly due to infections from HCV and hepatitis B virus (HBV), which are significant risk factors for the disease [178]. Recent studies have identified the PRLR in various human tissues, including the liver. When PRL binds to its receptor, it activates JAK2, a tyrosine kinase that subsequently phosphorylates STAT proteins. This activation plays a crucial role in cell proliferation and differentiation [179]. Research conducted by AbdelGhani et al. has shown that serum levels of PRL are significantly elevated in patients with HCC, suggesting that PRL could serve as a promising and potentially complementary biomarker for diagnosing this type of cancer [177]. A separate investigation into serum PRL levels among individuals infected with HCV revealed that these levels were significantly elevated compared to healthy controls. In particular, HCV‐infected males showed markedly higher PRL levels than their healthy counterparts, whereas the difference in levels among females was less pronounced [35].

In summary, PRL appears to have multifaceted roles in the context of HCV infection, influencing both hematological parameters such as thrombocytopenia and potentially contributing to immune dysregulation. Further research is needed to fully elucidate these relationships and their implications for treatment strategies (Table 4).

##### 5.1.1.5. Immunomodulatory Effects of PRL in Hepatitis B Infection

HBV is an enveloped DNA virus and serves as the prototype for the Hepadnaviridae family, which includes hepatotropic viruses. The viral genome measures approximately 3.2 kb in length and is encapsulated within nucleocapsids that possess an envelope. This structure is essential for the virus’s ability to infect liver cells and replicate efficiently within the host [202]. As noted by Shehata et al., there was a statistically strong significant difference in serum PRL levels between patients and controls. In particular, the serum PRL levels were significantly elevated in patients infected with HBV compared to those of healthy controls [180]. Giri et al. found that serum PRL levels in patients with acute viral hepatitis were similar to those in healthy controls. However, they observed that patients with liver cirrhosis exhibited significantly higher serum PRL levels, even in the absence of hepatic encephalopathy [181]. Ayfer reported that individuals with alcoholic and HBV‐related liver cirrhosis exhibited lower serum testosterone levels alongside elevated estradiol and PRL levels when compared to a control group. Furthermore, the study found a correlation between low testosterone and high PRL levels with the severity of cirrhosis [182]. NKCs play a crucial role in the innate immune response against HCV by utilizing mechanisms such as TRAIL and IFN‐γ. However, their functionality is significantly impaired in patients with chronic HCV infection (HCVp). PRL, an immunomodulatory hormone, has been shown to activate NKCs [183]. According to Medel et al., treatment with levosulpiride/cimetidine resulted in mild hyperprolactinemia, which correlated with enhanced NKC activation and a Th1‐type cytokine profile. Additionally, increases in TRAIL and IL‐2 were associated with reductions in viral load [183]. PRL levels are notably elevated in patients suffering from hepatic encephalopathy, a serious complication associated with liver disease. Research has shown that median PRL levels rise in correlation with the severity of encephalopathy, indicating a direct relationship between PRL concentrations and the deterioration of liver function. [184]. A serum PRL cutoff value of 50 ng/mL has been identified as a predictor of mortality for individuals with liver cirrhosis, particularly those experiencing hepatic encephalopathy. Elevated PRL levels have been linked to increased mortality rates, highlighting its potential role as a prognostic marker in liver disease [184, 185].

In summary, the observed elevation of serum PRL in patients with HBV infection most likely reflects hepatic injury rather than a direct viral effect on PRL synthesis or secretion. Studies have shown that cirrhotic patients whether of viral or alcoholic etiology exhibit a hormonal profile characterized by increased PRL and estradiol with reduced testosterone, similar to the endocrine pattern seen in pituitary neuroendocrine tumors (pitNET). This suggests that hepatic dysfunction per se disrupts dopaminergic regulation and systemic hormone clearance, leading to secondary hyperprolactinemia. Therefore, elevated PRL should be considered an indicator of liver damage severity and neuroendocrine dysregulation, rather than a biomarker of HBV activity [203]. Further mechanistic studies are warranted to clarify how liver pathology contributes to PRL alterations and whether these changes possess prognostic significance for cirrhotic disease progression (Table 4).

##### 5.1.1.6. Immunomodulatory Effects of PRL in Respiratory Syncytial Infection

Respiratory syncytial virus (RSV) is a major cause of lower respiratory tract illness in children [204]. A prospective cohort study involving 32 hospitalized infants diagnosed with RSV disease revealed a significant correlation between severe RSV infection and elevated serum PRL levels, as well as lymphopenia [186]. PRL interacts with its receptor (PRLR) on CD4^+^ T cells, contributing to altered Th1 polarization, and can modulate Treg by reducing their suppressive capacity [187, 190, 191]. Infants with the most severe disease manifestations show a distinct hormonal pattern of high levels of PRL and GH and reduced leptin correlating with clinical severity and intensive care admission [188, 189, 205–207]. Moreover, RSV‐specific IgA and lactoferrin in breast milk, under PRL influence, may enhance mucosal maturation [206, 207].

In summary, the interplay between PRL and RSV is complex, encompassing both proinflammatory and anti‐inflammatory mechanisms. Elevated levels of PRL during RSV infections may exacerbate the severity of the disease by influencing immune responses. A deeper understanding of this relationship could lead to the development of therapeutic strategies aimed at modulating PRL’s effects on viral infections (Table 4).

##### 5.1.1.7. Immunomodulatory Effects of PRL in SARS‐CoV‐2 Virus Infection

The COVID‐19 pandemic, caused by SARS‐CoV‐2, affects multiple organs, including the endocrine system [204, 208–212]. This diverse range of symptoms is largely attributed to the widespread presence of the angiotensin‐converting enzyme 2 (ACE2) receptor throughout the human body, which is believed to serve as a critical entry point for SARS‐CoV‐2. The binding of the virus to the ACE2 receptor is a crucial step in its cellular entry mechanism and requires the involvement of transmembrane serine protease 2 (TMPRSS2) for the priming of the viral spike glycoprotein [213]. The stress induced by COVID‐19 may also influence the secretion of PRL and other hormones related to stress responses [192].

Evidence indicates that SARS‐CoV‐2 infection may raise PRL levels [193–195, 214, 215], and moderate increases appear beneficial to immune recovery, whereas excessive levels enhance cytokine‐driven inflammation [91].

PRL interacts with sex hormones and pituitary function. Disruption of gonadal hormones and a significant rise in serum PRL levels have been observed in male patients infected with SARS‐CoV‐2 [215, 216]. Elevated levels of PRL can inhibit the function of the pituitary gland, leading to a decrease in gonadotropin production [217]. Notably, women maintain elevated levels of PRL even after menopause, which does not decrease with age. This persistent elevation in PRL levels among women may contribute to the observed gender bias in COVID‐19 outcomes, suggesting that biological factors beyond hormonal changes could be influencing survival rates during the pandemic [208–212, 218, 219]. An H2 receptor antagonist has been shown to alleviate symptoms in COVID‐19 patients, although the exact mechanism remains unclear [220]. It is noteworthy that, similar to dopamine antagonists, H2 blockers also elevate blood PRL levels [221]. Sharifzak et al. conducted a study that found higher levels of malondialdehyde (MDA) across all time intervals measured, as well as increased NO levels at the first time interval in females compared to males. This research suggests a significant relationship between hyperprolactinemia and elevated lipid peroxidation, alongside increased NO production [210]. In summary, the relationship between PRL levels and COVID‐19 outcomes underscores a dual role where elevated serum PRL can either protect against or worsen the effects of the virus, depending on the infection phase and the patient’s underlying health conditions. A cohort study involving 30 men and 15 women who underwent the Trier Social Stress Test indicated a significant increase in PRL levels in response to stressors [196], potentially linked to the COVID‐19 pandemic [5]. Treg and proinflammatory pathways such as Toll‐like receptor 4 (TLR4) and NF‐κB activation are also modulated by PRL [222]. A study examining men with COVID‐19 found elevated levels of PRL and LH, alongside reduced testosterone and follicle‐stimulating hormone (FSH) levels. These hormonal changes suggest the presence of primary testicular damage occurring during the active phase of the disease [197]. Pregnant women display mild symptoms, possibly benefiting from PRL‐related immune support [223]. Alterations in the endocrine cells of the adenohypophysis have been observed in patients infected with SARS‐CoV. These changes correlate with increased serum levels of PRL, FSH, and LH, alongside decreased serum levels of GH, TSH, and ACTH [224]. Similar hormonal disturbances have been noted in patients infected with SARS‐CoV‐2, particularly concerning LH, PRL, GH, and TSH [194, 225]. Obesity and low PRL levels correlate with worse prognoses [91, 226]. Cigarette smokers exhibit higher PRL after nicotine exposure, which may partly dampen cytokine storms [91, 227, 228]. Another investigation highlighted a moderate positive correlation between PRL and CRP, an inflammation marker, indicating that increased PRL levels may be associated with heightened inflammatory responses in severe COVID‐19 cases [229]. The precise mechanisms by which PRL affects COVID‐19 outcomes are not fully understood. It is suggested that PRL may play a role in enhancing hyperinflammatory responses by promoting the production of proinflammatory cytokines, while simultaneously having anti‐inflammatory effects that could help reduce excessive inflammation [4, 50].

It should be noted that PRL is not the sole determinant of the stronger and more sustained immune responses observed in females. Recent genomic and epigenetic discoveries have revealed additional biological mechanisms underlying this sexual dimorphism. One crucial factor is the escape of the TLR7 gene from X‐chromosome inactivation in females, leading to enhanced Type I interferon responses and contributing both to protection against viral infections and to the higher prevalence of autoimmune diseases among women [230]. Furthermore, dysregulation of XIST, the master X‐inactivation RNA, has been directly implicated in human autoimmunity, promoting overexpression of X‐linked immune genes [231]. Together with hormonal factors such as estrogens and progesterone, these pathways illustrate that immune sexual dimorphism is a multifactorial phenomenon in which PRL acts as one, but not the only, contributing element.

In conclusion, PRL seems to have a crucial role in the immune response to COVID‐19, as higher levels are associated with increased disease severity and inflammatory markers. Nevertheless, additional research is necessary to elucidate its specific functions and mechanisms related to SARS‐CoV‐2 infection, as well as to assess the long‐term effects on endocrine health in patients who have recovered (Table 4).

##### 5.1.1.8. Immunomodulatory Effects of PRL in Influenza Infection

Four types of influenza viruses exist in nature: Influenza A, B, C, and D. Although Influenza A, B, and C can infect humans, only Influenza A and B cause annual seasonal epidemics. Influenza D is primarily found in pigs and cattle and does not currently cause human illness. Among these types, Influenza A viruses are the most significant for human health, responsible for widespread morbidity, mortality, and at least five documented pandemics throughout the twentieth century [232]. Gene regulation analysis in the mammary gland demonstrated downregulation of milk production genes, including PRL, suggesting a potential viral interference mechanism disrupting lactation processes during influenza infection [198]. A novel metabolite originating from PRL, which shares structural similarities with GH, demonstrates potential therapeutic efficacy in combating severe Influenza A virus infections [199]. H1N1 influenza infection leads to a significant downregulation of milk production genes, including PRL [198] (Table 4).

## 6. Therapeutic Implications of PRL

### 6.1. Targeting PRL in Viral Infection Treatments

Viral infection treatments may benefit from PRL’s intricate immunological properties, as this hormone demonstrates nuanced potential for modulating immune responses and developing targeted therapeutic interventions. PRL enhances NKC activation, extends T lymphocyte viability, and stimulates IFN‐γ synthesis, thereby modulating critical immune system responses [5, 50]. The compound exhibits a complex immunomodulatory profile, simultaneously triggering proinflammatory cytokine production while also demonstrating the capacity to suppress inflammatory responses, thus presenting a multifaceted and context‐dependent regulatory mechanism [4, 5].

### 6.2. Potential for PRL Modulators in Clinical Use

PRLR modulators demonstrate promising potential for cancer treatment, particularly in breast and prostate cancers. Research indicates that PRLR signaling plays a critical role in tumor progression, making it an attractive therapeutic target [13, 233]. Emerging scientific evidence indicates that PRL may serve as a nuanced immunomodulatory agent, demonstrating the potential to regulate and potentially attenuate proinflammatory cascades, particularly in complex physiological contexts such as human parturition [234]. Scientists are investigating PRLR modulators as an innovative therapeutic strategy, acknowledging the critical importance of developing alternative interventions to address PRL‐related clinical disorders [235].

### 6.3. Limitations of PRL as a Disease Biomarker

Recent evidence does not support the use of PRL as a biomarker for viral illness. Large‐scale proteomic datasets from the Human Protein Atlas have shown that elevated circulating PRL levels are primarily associated with pitNET, certain central nervous system neoplasms, and psychiatric disorders secondary to antidopaminergic drug administration. No consistent elevation has been found in common viral infections [203]. Therefore, although PRL may fluctuate in individual viral cases, current population‐based data indicate that PRL cannot serve as a reliable or disease‐specific marker for viral pathology. Its role in immune activation should thus be interpreted strictly as mechanistic rather than diagnostic.

## 7. Conclusion

PRL serves as a pivotal molecular messenger, intricately linking the endocrine and immune systems through its nuanced and multidimensional biological roles, underscoring the profound complexity of physiological interactions and emphasizing the critical importance of ongoing scientific investigation to elucidate its sophisticated immunomodulatory pathways.

### 7.1. Future Directions and Research Opportunities

Further rigorous experimental and clinical investigations are imperative to elucidate the multifaceted mechanisms by which PRL interacts with viral pathogenesis, exploring its nuanced potential as a sophisticated diagnostic biomarker and innovative therapeutic intervention strategy across diverse viral disease landscapes.

**Nomenclature**
ACE2: Angiotensin‐converting enzyme 2
ACTH: Adrenocorticotropic hormone
AITD: Autoimmune thyroid diseases
ALV‐J: Avian leukosis virus
APC: Antigen‐presenting cell
ART: Antiretroviral therapy
CIS: Clinically isolated syndrome
CRP: C‐reactive protein
CVB: Coxsackieviruses B
CypA: Cyclophilin A
DDC: Dopamine decarboxylase
FSH: Follicle‐stimulating hormone
GH: Growth hormone
HBV: Hepatitis B virus
HCC: Hepatocellular carcinoma
HCMV: Human cytomegalovirus
HCV: Hepatitis C virus
HIV: Human immunodeficiency virus
hPRL: Hypergammaglobulinemia
IFN: Interferon
IL: Interleukin
iNOS: Inducible nitric oxide synthase
IP‐10: Interferon‐gamma protein 10
IRF‐1: Interferon regulatory factor‐1
JAK2: Janus kinase 2
LH: Luteinizing hormone
LPS: Lipopolysaccharide
MAPK: Mitogen‐activated protein kinase
MCP: Monocyte chemoattractant protein
MIP: Macrophage inflammatory protein
MS: Multiple sclerosis
NF‐κB: Nuclear factor kappa‐light‐chain‐enhancer of activated B cells
NK: Natural killer
NKC: Natural killer cell
NMO: Neuromyelitis optica
NREM: Nonrapid eye movement
PBMC: Peripheral blood mononuclear cell
PIF: Primary physiological prolactin inhibitory factor
PK: Protein kinase
PREB: PRL regulatory element binding
PRF: Prolactin‐releasing factor
PRL: Prolactin
PRLR: Prolactin receptor
PTK: Protein tyrosine kinase
RA: Rheumatoid arthritis
ROS: Reactive oxygen species
RRMS: Relapsing‐remitting multiple sclerosis
RSV: Respiratory syncytial virus
SARS‐CoV: Severe acute respiratory syndrome coronaviruses
SLE: Systemic lupus erythematosus
STAT: Signal transducer and activator of transcription
T3: Triiodothyronine
Th: T‐helper
TIDA: Tuberoinfundibular dopamine
TLR4: Toll‐like receptor 4
TMPRSS2: Transmembrane serine protease 2
TNF‐α: Tumor necrosis factor‐alpha
Treg: Regulatory T
TRH: Thyrotropin‐releasing hormone
TSH: Thyroid‐stimulating hormone

## Conflicts of Interest

The authors declare no conflicts of interest.

## Funding

This study was funded by Kerman University of Medical Sciences (404000880).

## Data Availability

Data sharing is not applicable to this article as no datasets were generated or analyzed during the current study.
